# Predictors of quality of childcare centers in low-income settings: findings from a cross-sectional study in two Nairobi slums

**DOI:** 10.3389/fpubh.2023.1163491

**Published:** 2023-10-31

**Authors:** Margaret Nampijja, Nelson Langat, Linda Oloo, Kenneth Okelo, Ruth Muendo, Martin Kiyeng, Patrick Amboka, Mary Abboah-Offei, Anna Ray, Patricia Kitsao-Wekulo, Elizabeth W. Kimani-Murage, Helen Elsey

**Affiliations:** ^1^African Population and Health Research Centre, Nairobi, Kenya; ^2^Kidogo Early Years, Nairobi, Kenya; ^3^School of Health and Social Care, Edinburgh Napier University, Edinburgh, United Kingdom; ^4^Department of Health Sciences, University of York, York, United Kingdom

**Keywords:** predictors, quality, nurturing care, childcare centers, slums, informal settlements

## Abstract

**Background:**

Rapid urbanization and increased women’s involvement in paid work have contributed to the upsurge of informal childcare centers, especially in low-income settings where quality is a major issue. However, there are limited data on the factors associated with the quality of childcare centers in informal settlements in Africa.

**Methods:**

We conducted a quantitative observation and questionnaire survey of 66 childcare centers to identify the factors associated with the quality of childcare services in two informal settlements (Korogocho and Viwandani) in Nairobi. The quality of the centers (outcome variable) was assessed using a locally developed tool. Data on center characteristics including type, size, location, length of operation, charges, and number of staff were collected. Center providers’ knowledge, attitude, and practices (KAP) in childcare were assessed through a questionnaire, focusing on nurturing care and business management. Data were described using means and standard deviation or frequencies and percentages. Associations between quality center score (outcome variable) and other variables were examined using multivariable linear regression to identify potential predictors of the quality of the center environment.

**Findings:**

A total of 129 childcare centers were identified and categorized as home-based (*n* = 45), center-based (*n* = 14), school-based (*n* = 61), and church-based (*n* = 9). The number of home-based centers was particularly high in Viwandani (*n* = 40; 52%). Only 9% of home-based centers reported any external support and 20% had any training on early childhood development. Of the 129 centers, 66 had complete detailed assessment of predictors of quality reported here. Unadjusted linear regressions revealed associations between quality of childcare center and center providers’ education level, type of center, support received, caregiver–child ratio, number of children in the center, and center providers’ KAP score (*p* < 0.05). However, in the multivariable regression, only higher levels of center provider KAP ( β = 0.51; 95% CI: 0.18, 0.84; *p* = 0.003) and center type ( β = 8.68; 95% CI: 2.32, 15.04; *p* = 0.008) were significantly associated with center quality score.

**Implication:**

Our results show that center providers’ knowledge and practices are a major driver of the quality of childcare centers in informal settlements in Nairobi. Interventions for improving the quality of childcare services in such settings should invest in equipping center providers with the necessary knowledge and skills through training and supportive supervision.

## Background

There is a growing focus on early childhood development (ECD) in low- and middle-income countries (LMICs) with the World Health Organization’s (WHO) Nurturing Care Framework (1), providing a much-needed guide to improve early childhood health, nutrition, safety, early learning, and development. Governments and donor organizations have supported mainstream ECD programs and policies, particularly on health and nutrition and pre-school-age children (older than 3 years) (2).

Despite the challenges, substantial efforts have been made to improve childcare in sub-Saharan Africa. Governments, NGOs, and international organizations have launched various programs and policies to address the needs of children. The African Union’s Agenda 2063 emphasizes investing in early childhood development and recognizing its long-term impact (3). Additionally, the UNICEF-led Early Childhood Development Action Network (ECDAN) has been instrumental in expanding access to quality childcare services across the region (4, 5).

These studies have alienated challenges with childcare and how financing childcare through subsidies can enable women to engage in paid work to earn; however, there is little focus on childcare services for younger children (0–3 years) in LMICs (6). Sub-Saharan Africa grapples with several challenges in providing adequate childcare. Factors such as poverty, limited access to education, and healthcare disparities contribute to suboptimal childcare conditions. High child mortality rates, malnutrition, and inadequate early childhood development opportunities remain significant concerns. Childcare for 0–3 years in LMICs is almost exclusively provided by the private sector, and in low-income communities, the quality leaves a lot to be desired.

The need for childcare services became critical in East and Central Africa with the advent of the HIV scourge, which left several children without parents (primary caregivers) and led to the establishment of alternative childcare services including institutional- and community-based care, such as foster care by relatives and others. However, as revealed by Save the Children, UK, in the majority of these childcare services, the quality was below the standard stipulated in the UN Convention on the Rights of Child (UNRC). Core to the poor quality is the lack of understanding of the nature of care that is tailored to a non-family care context. To address this gap, Save the Children, UK, working with other partners, put together a comprehensive set of quality indicators that can be applied across diverse contexts including resource poor and emergency settings, to guide the assessment and improvement of childcare for child development. The focus on childcare is particularly important in the context of rapid urbanization, with over half of the world’s population living in urban settings and increases in women’s employment outside the home. This has contributed to an increasing demand for affordable childcare options, particularly in low-income urban settings.

Quality childcare centers have the potential to provide multiple benefits to children, families, and societies (1) through women’s participation in the labor force (7–11). Increases in parental employment, particularly of mothers, have the potential to provide indirect benefits to the children through increased household income and improved nutrition (12). A well-facilitated childcare center that provides opportunities for learning and play, good feeding, and access to healthcare has the potential to nurture and optimize child development (13–18) with stronger benefits for children living at an economic disadvantage (19). WHO’s Nurturing Care Framework specifies the need for an environment that promotes children’s good health, appropriate nutrition, responsive caregiving, safety and security, and opportunities for early learning.

The *Good health* component of the nurturing care framework ensures the health and wellbeing of the child and caregiver and includes family planning, HIV testing, prevention of mother-to-child transmission of HIV, essential newborn care including kangaroo care for small babies, immunization of mother and child, growth monitoring and counseling, promotion of health and wellbeing including healthcare-seeking behavior, prevention and treatment of childhood illness, and caregiver’s physical and mental health problems, and care for children with developmental difficulties or disabilities. The *Adequate nutrition* component emphasizes good mother’s nutrition during pregnancy, exclusive breastfeeding (0–6 months), balanced complementary feeding and weaning from 6 months, food safety and family food security, and feeding practices that accommodate social and emotional interaction.

*Responsive caregiving* emphasizes observing and responding to children’s movements, sounds and gestures, and verbal requests. It also highlights the importance of mutually enjoyable interactions to create an emotional bond, which helps young children understand the world around them and learn about people, relationships, and language.Responsive caregiving is thus the basis for protecting children against injury and the negative effects of adversity, recognizing and responding to illness, enabling enrichment, and building trust and social relationships.

*Opportunities for early learning* is based on the fact that learning begins from conception, and hence, this component emphasizes the importance of providing opportunities for children to acquire skills and capacities interpersonally, in relationship with other people, through smiling and eye contact, talking and singing, modeling, imitation, and play.

*Safety and security* emphasizes the need to ensure a safe and secure environment for children, protection from physical harm/injury and emotional/psychological stress (fear and anxiety) and maltreatment, and ensuring good mental health of the caregiver.

The five nurturing care components together provide for the provision of quality childcare services (1) and the criteria for aspects to consider while assessing the quality of childcare centers and other environments in which children are raised.

The importance of ensuring quality provision of childcare cannot be overstated with evidence of poor quality childcare centers that provide limited cognitive stimulation being likely to limit children’s development (20). However, worldwide, more than 40% of all children below primary school age or nearly 350 million in LMICs do not have access to the quality childcare services they need (20). Inequalities in childcare provision are worse in specific parts of the world, and they certainly deepened significantly following the lockdown measures to control the COVID-19 pandemic which resulted in the closure of already limited childcare services. Children are exposed to poor-quality, home-based, and center-based childcare that increases the risk of poor outcomes (21). Such low-quality childcare services are reported across the diversity of socioeconomic divides within Africa. In East and Central Africa, despite the increased awareness of and intention to support children’s rights on the part of individuals, NGOs, and governments, the majority of institution- and community-based childcare services are still not meeting the desired standard (Save the Children, UK report) (22). This is mostly in the poorest communities such as the urban informal settlements where poverty, high illiteracy levels, poor infrastructure, and lack of access to services contribute to poor quality (23). In the more developed countries, e.g., South Africa, there has been significant progress in childcare and ECD services, with approximately 58% of children accessing childcare; however, there are still gaps in infrastructure, nutrition, ECD programs, teacher training, institutional capacity, and funding, and the major drivers are poverty, education, health, and HIV/AIDS (24). Similar challenges are reported in low-income settings outside Africa (25). Beyond the influence of poverty, unique and contextually specific factors appear to determine the quality of childcare centers across low-income settings (26, 27). For instance, in Florida, USA, the best predictors of higher quality care and sensitive caregiver–child interaction in centers were specialized caregiver training, higher adult–child ratios, use of planned activities, and less perceived stress by caregivers (28). There is, however, limited empirical data on the contextual drivers of the quality of childcare centers in urban–poor (slum) communities in sub-Saharan Africa. We aimed to establish the quality of childcare centers in two slums in Nairobi, Kenya, and identify key factors associated with quality (including knowledge and skills of center providers, characteristics of the childcare centers, and the center providers’ sociodemographic characteristics). This assessment preceded the co-design and feasibility testing of an intervention that aimed to improve the quality of childcare service provision in low-income urban neighborhoods.

## Methods

### Study design

The current study forms a part of a larger study that employed a phased sequential feasibility design with pre- and post-intervention assessments of the quality of childcare environment in two informal settlements (Korogocho and Viwandani) in Nairobi (29). Baseline (pre-intervention) assessments reported in this study were based on a cross-sectional survey to map and profile childcare centers in the two settlements prior to the implementation of the intervention.

### Study setting

The study was conducted in Korogocho and Viwandani, two large informal settlements in Nairobi. These communities represent urban poor settings where children of working mothers are usually taken to local low-cost childcare centers, which are likely to provide substandard care. The sociodemographics of these communities have been well characterized within the Nairobi Urban Health and Demographic Surveillance System (NUHDSS) by the African Population and Health Research Centre (APHRC) (23). Korogocho and Viwandani slum settlements, located approximately 7 km from each other, are densely populated with 63,318 and 52,583 inhabitants per square km, respectively. The settlements are characterized by poor housing, poor sanitation, lack of basic infrastructure, insecurity, high crime rate, and poor access to maternal and child health (MCH) services and healthcare in general (23). A high proportion of mothers in these communities are engaged in low-paid employment, which only affords low-quality childcare within the community.

### Participant and inclusion criteria

**Figure 1:**
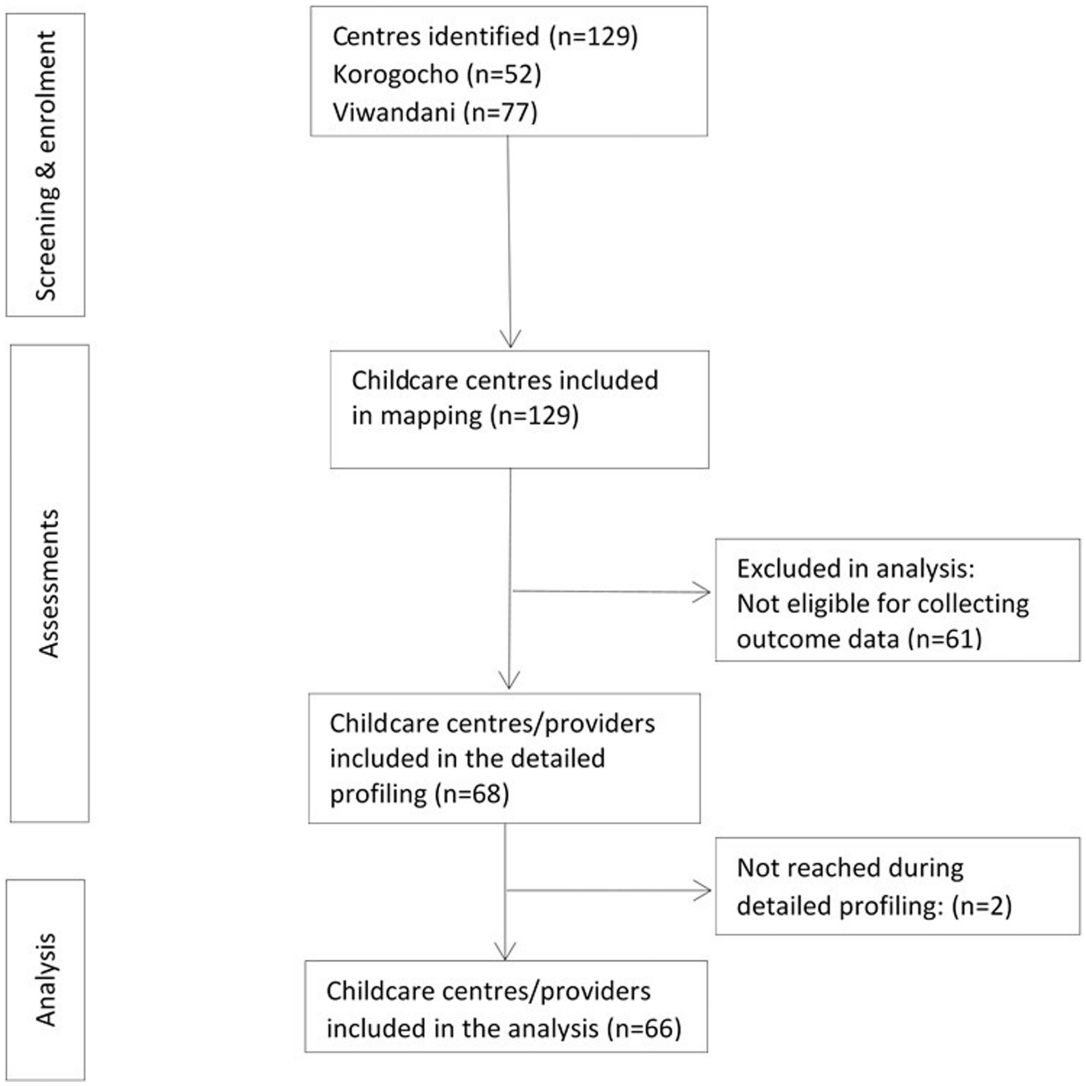
Flow diagram of childcare centers/providers from identification to analysis.

Centers were eligible for mapping and profiling if they were located in either one of the two settlements, if they provided paid childcare services for children under five 5 years and were operational, and if the managers/owners were willing to participate in the study. We focused on children younger than 5 years because they do not go to pre-school and their parents or guardians are likely to use childcare services when they go outside for work. By design, all eligible childcare centers within Korogocho and Viwandani were included in the mapping and profiling exercise, except for those that were not available after three visits by the data collectors. We identified 129 childcare centers, 52 in Korogocho and 77 in Viwandani, that were operational at the time of the data collection. Eligibility for the detailed quality assessment was that the center was home-based, center-based, or faith-based as defined in Table 1. Due to the relatively high levels of support provided to school-based centers, these were not included in the detailed assessment and intervention. Based on this criterion, 68 out of 129 centers were eligible for detailed assessments, of which 66 had complete data (Figure 1). These centers form the study sample for the current study.

**Table 1:** Typology of center-based childcare providers.

Home-based	These centers are within the dwelling units of the childcare center providers. In some cases, the providers hired separate rooms within the location where they lived and designated the rooms for use as childcare centers. In most cases, however, the same room where the childcare center provider lived also served as the childcare center. Most of the center providers in this category started the childcare center business out of necessity (due to lack of employment) and initially started by looking after neighbors’ children and then turned this into business. The majority do not have any training in ECD or in any other childcare-related aspects.
School-based	These centers are based on the school system, usually attached to a primary school. In these cases, the schools have a childcare center unit, which also serves as a pre-primary school unit. Most of the care providers in this category are trained ECD and primary school teachers; some are also pursuing degrees in education.
Center-based	These are autonomous centers operating in buildings, purposely built for the provision of childcare services. They are not a part of a residential building and do not have a primary school section. Some of the providers in this category are ECD-trained.
Faith-based	These are childcare centers that are nested within a church or a mosque and are started by the church/Mosque. The teachers are also employed by the respective faith groups.

### Procedures

#### Mapping of childcare centers

Childcare centers in Korogocho and Viwandani informal settlements in Nairobi County were mapped using the Global Positioning System (GPS) coordinates. Residents from the two settlements were recruited and trained as field interviewers (FIs) to conduct the mapping survey. These FIs had a minimum of O-level education, were fluent in Kiswahili, were familiar with the study community, and had some experience in administering qualitative and quantitative interviews. Within their designated villages and with the support of community health volunteers (CHVs), FIs visited households asking if there were any centers where staff were paid to look after children younger than 5 years. Childcare centers in nine villages in Korogocho (Grogan A, Grogan B, Gitathuru, Nyayo, Kisumu Ndogo, Korogocho A, Korogocho B, Highridge, and Ngomongo), covering 0.86 square kilometers, and in seven villages in Viwandani (Paradise, Sinai, Jamaica, Lunga Lunga, Donholm, Kingstone, and Riverside), covering 0.59 square kilometers, were mapped. Once the FIs identified a childcare center, they captured their location details using GPS (embedded in the SurveyCTO platform that was used for data collection). Then, they recorded basic details of the center through interviews with the childcare providers. The process included observing the facilities in the childcare center and checking any available records on basic information on the childcare centers, e.g., length of operations, opening hours, numbers of staff, children, rooms, age of children (upper and lower limits), charges (per day and any additional time/out of hours), type of center, any organizational support, and name of local CHVs to enable the delivery of the intervention in the implementation feasibility phase. A typology of childcare providers was developed by the investigator team together with Kidogo, a social enterprise, that runs childcare centers in informal settlements in Nairobi and is a partner in this study, and the sub-county health team to enable categorization of the assessed centers (Table 1). Since the number of childcare centers in Korogocho and Viwandani was not known, we aimed to include as many centers as possible, including the different types of childcare centers. Childcare center providers were asked if they were interested in more detailed quality assessments and knowledge, skills, and attitude assessments using questionnaires. Those who gave consent to a detailed assessment and joined the proposed skills-building CoP intervention were included in the detailed assessments that were conducted after 2–3 weeks of the mapping.

#### Assessing the quality of the childcare environment

Following the mapping, the field team went back to the mapped childcare centers that agreed to be assessed to conduct a detailed assessment of center quality and skills of center providers and CHVs. A quality assessment tool was developed, drawing on tools currently used by Kidogo. Tools, such as the Family Childcare Environment Rating Scale®, Revised (FCCERS-R) (30), have been considered for use; however, as many of such tools were developed and used in high-income contexts, items required considerable adaptation to the context of informal settlements in Kenya. Furthermore, we planned to design the tool to be used within routine practice by a CHV or other community workers, to support the improvement in the childcare centers. We anticipated that the simple quality assessment tool would be revised during the co-design and implementation process, as we learned more about its feasibility, appropriateness, and the type of information required. The quality assessment tool benefited from feedback received from sub-county health teams and parents and center providers themselves on the weak areas in childcare, together with the materials used by Kidogo. The tool focused on (i) child protection, safety, discipline, and abuse; (ii) stimulating environment; (iii) responsive caregiving; (iv) learning through play; (v) health; (vi) nutrition; (vii) water, sanitation, and hygiene; and (viii) business and administration. With the exception of business administration, the domains align with the Nurturing Care Framework components of the WHO, i.e., good health, adequate nutrition, responsive caregiving, safety and security, and opportunities for early learning (1). The business domain focuses on the capability of center providers to provide quality service while at the same time earning an income to live. Several items were generated under each category. They were piloted at a few centers and refined further, yielding 38 items that were used in the assessments. Each item is scored by ticking in the box against the item if available or by crossing in the box if the item is not available. Each ticked item was equivalent to one score, and the total score was the number of all the ticked items. The total score was converted to a percentage to enhance intuition. A combination of interviews and observations done within the center was used to administer the tool. On average, the tool took approximately 45 min to complete. Details of the tool are presented in Appendix 1.

#### Assessing knowledge, skills, and attitudes of childcare center staff

Questionnaires were administered to the childcare providers to assess their knowledge, skills, attitudes, and opportunities/barriers to implement this knowledge and attitudes within the areas of stimulation, nutrition, health and safety, staff and training, parent involvement, and resource management. The assessment was administered to center providers who agreed to the quality assessment visits. Similar to the quality assessment tool, the skills questionnaire focused on: (i) child protection, safety, discipline, and abuse; (ii) stimulating environment; (iii) responsive caregiving; (iv) learning through play; (v) health; (vi) nutrition; (vii) water, sanitation, and hygiene; and (viii) business and administration. The items were piloted on approximately 10 center providers and finalized with 53 items. Details of the tool are presented in Appendix 2. The interviews were conducted face-to-face or on the phone for those who could not be reached because of the movement restrictions due to COVID-19. Interviews lasted, on average, for 45 min to 1 h.

### Data analysis

#### Descriptive analysis

Descriptive statistics (frequencies, means, medians, standard deviations, and interquartile ranges) were used to summarize the data. Means (SD)/median (IQR) were reported for continuous variables such as the quality of care score, while frequencies and percentages were used for the categorical variables such as type of childcare center. Scatter plots with fitted values were used to illustrate the degree and direction of the relationship between the quality of care score and each of the continuous independent variables: center provider age, KAP score, number of children in the center, years of operation of childcare center, and the caregiver/child ratio. In addition, Pearson’s correlation was used to quantify the degree of the relationship between the quality of childcare center and potential predictive factors. Some continuous variables (number of children, years of operation, and caregiver-to-child ratio) were log-transformed to improve their linear relationship with the dependent variable (quality of care score). The correlation between predictor variables (center provider KAP score, age, education level, caregiver–child ratio, and years of operation) was determined using Spearman’s rank correlation since an ordinal variable (education level) was involved. Mean (95% CI) quality of childcare center scores were plotted against the categorical predictors (center provider sex, location of childcare center, level of education of provider, and the type of childcare center).

#### Analysis for predictors of quality of childcare centers

The association between the quality of childcare center score (outcome) with predictors was examined using linear regression. Simple linear regression and multiple linear regression with robust standard errors were used to determine the crude and adjusted associations, respectively, between the quality of care score and the predictors. In the models, the dependent variable was the quality of care score, while the independent variables included center provider knowledge and skills score (%), center provider sociodemographic characteristics, and childcare center characteristics, such as type, location, size, and duration since establishment. The β -coefficient and the corresponding 95% confidence interval and value of p were reported. Mapping data (GPS coordinates) were used to generate maps to display the distribution of childcare centers within the two locations by type and offset to maintain the anonymity of centers.

## Results

### Basic mapping and profiling characteristics of childcare centers

**Figure 2:**
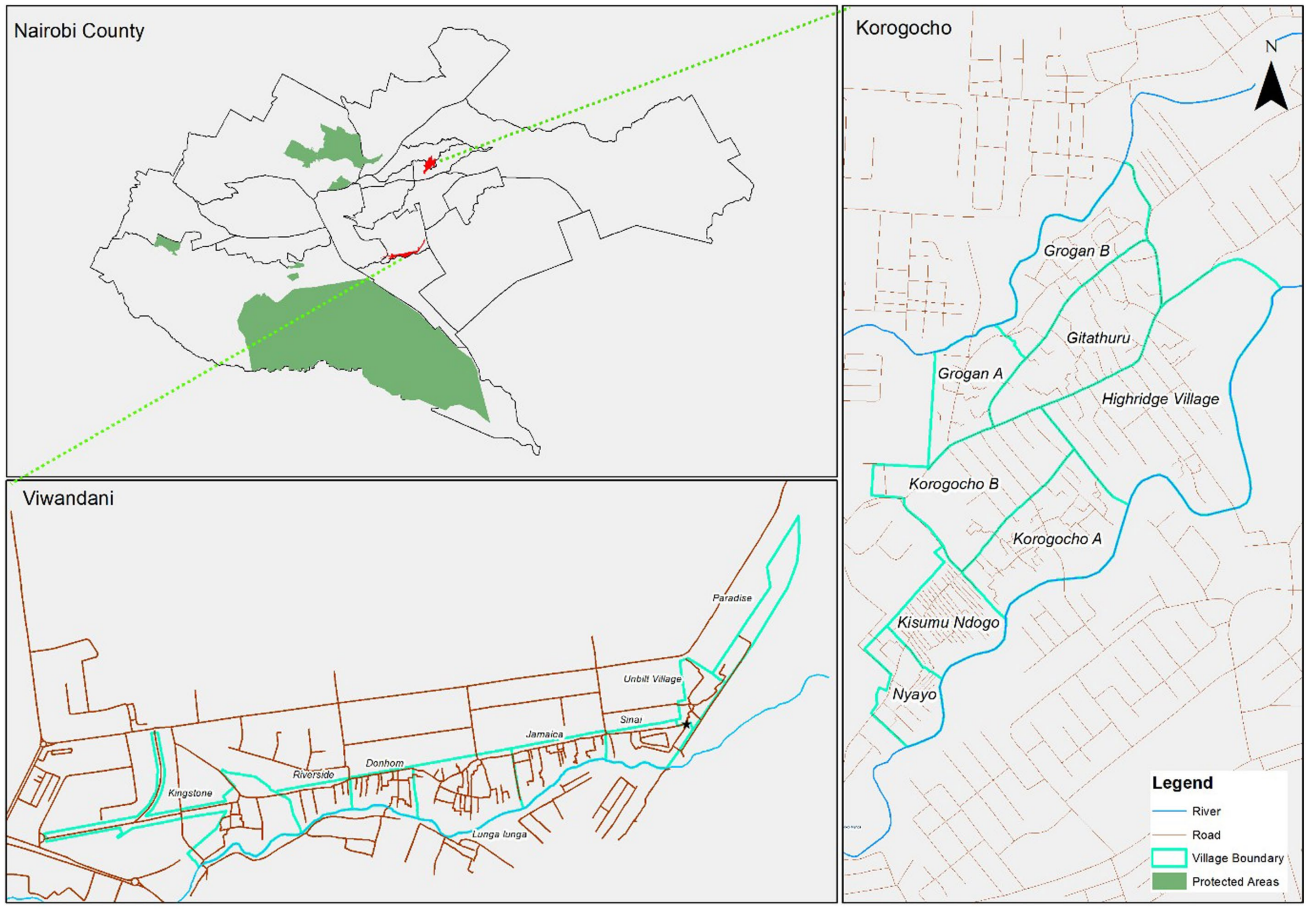
Base maps – Nairobi County ward boundaries and the two study areas.

**Figure 3:**
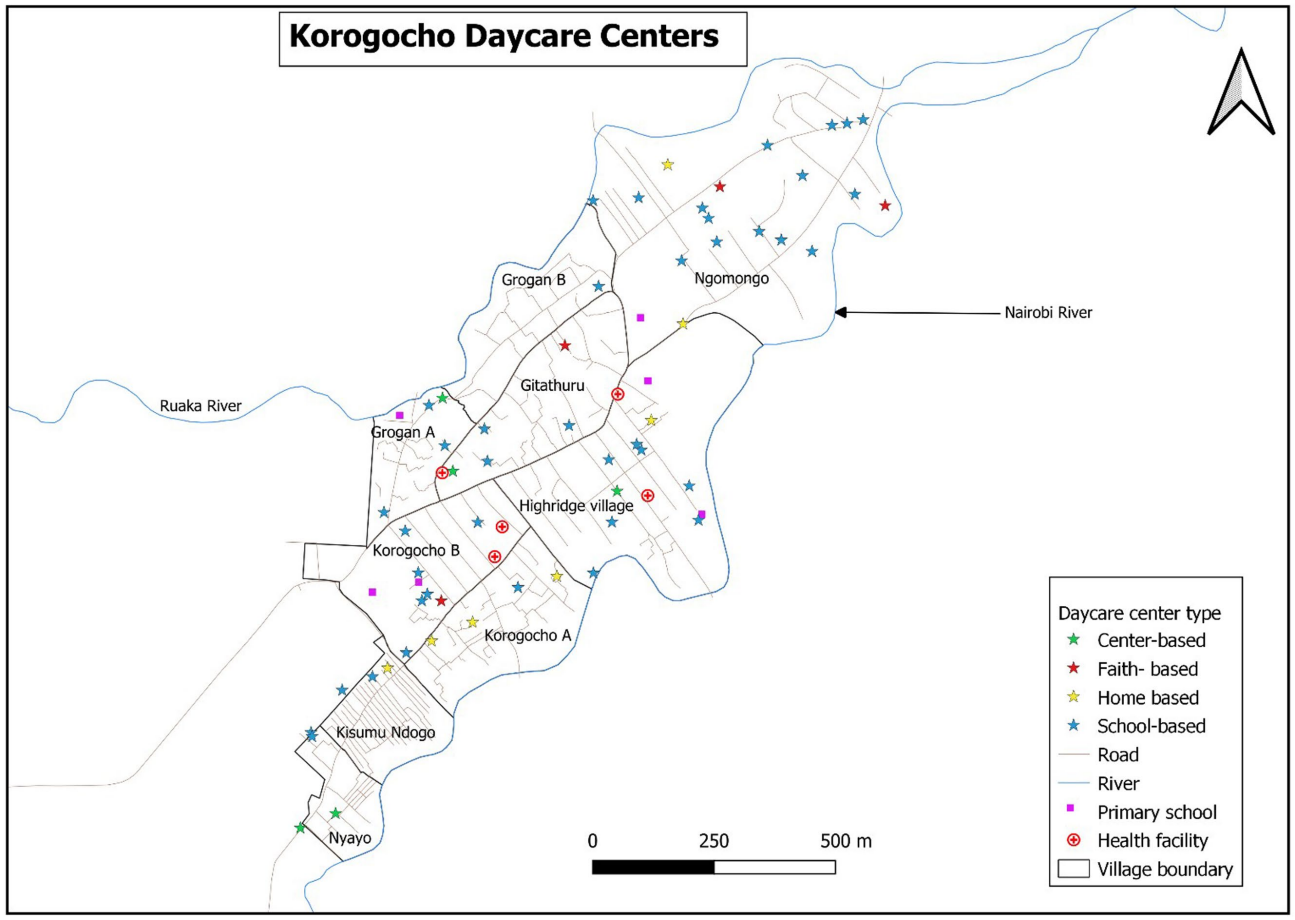
Distribution of the childcare centers by type and the essential facilities in Korogocho.

A total of 129 informal childcare centers (defined as childcare centers within the slums) were identified in Korogocho and Viwandani slums. A significant proportion of the identified centers (40%) was attached to schools; however, several others (35%) were home-based. This was particularly the case in Viwandani where 52% of all mapped centers were home-based. Home-based center providers reported particularly low levels of support (9%) and training on ECD (20%) compared with the centers, faith, or school-based centers. The distribution of these centers in the two study sites is presented in Figures 2, 3.

**Figure 4:**
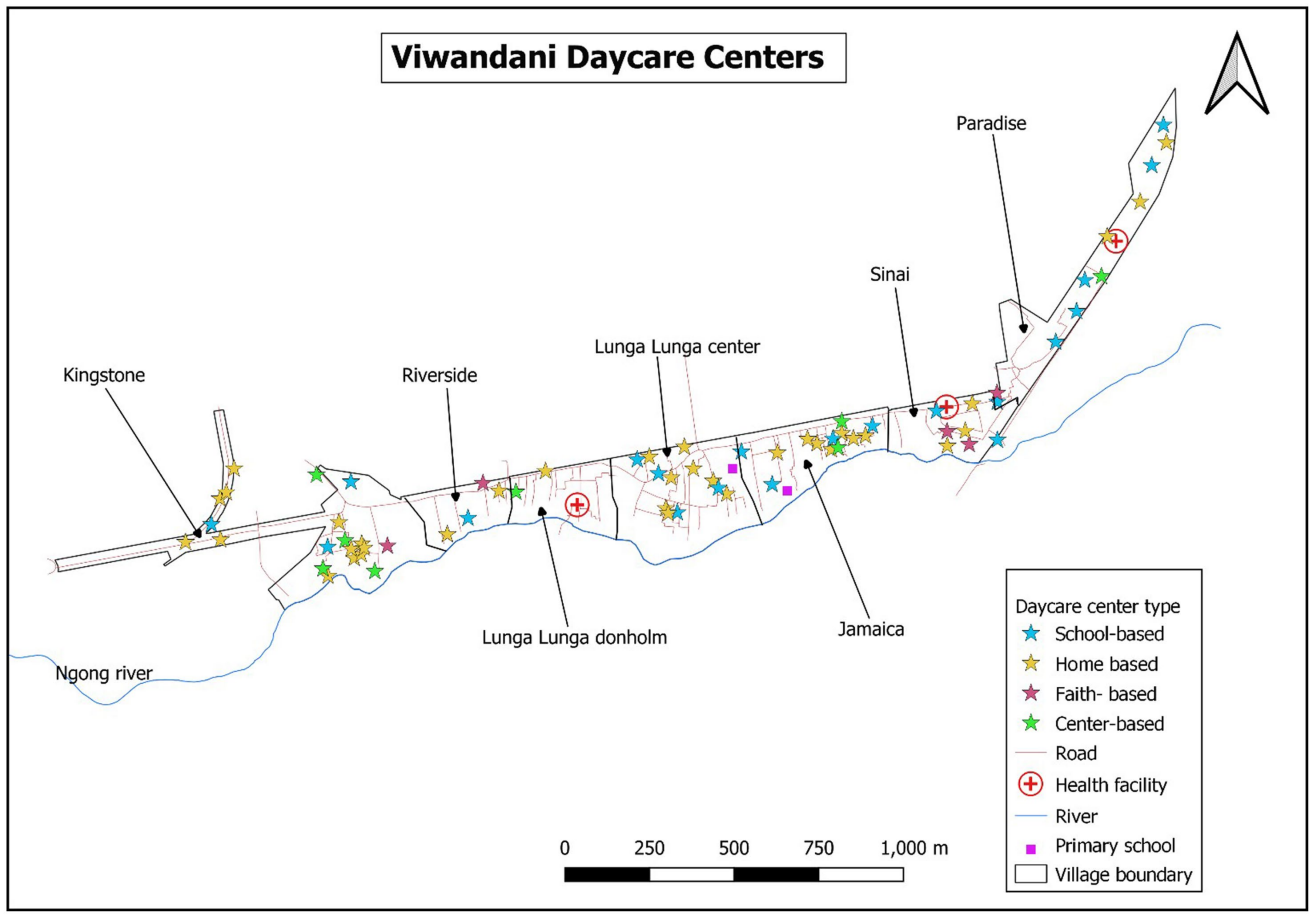
Distribution of the childcare centers by type and the essential facilities in Viwandani.

The map of Nairobi County with ward boundaries puts into context the location of the two study areas located in different wards (Figure 2). As shown by maps in Figures 3, 4, there was a higher concentration of home-based centers than other types in Viwandani, while in Korogocho, the school-based centers were more dominant than the other types. Within each location, the centers were not evenly distributed across the area, and they tended to cluster along the roads and junctions.

### Detailed quality assessment

A total of 66 childcare centers were profiled in Korogocho and Viwandani settlements in Nairobi between 25 March 2021 and 13 April 2021. More than three-quarters (77%) of the childcare centers were in Viwandani, and almost all (95%) of the center caregivers were women (Table 2). The mean age of the center caregivers was 40 years, ranging from 23 to 74 years. Approximately two-thirds (68%) of the centers were home-based, while the median of caregiver: child ratio was 8, ranging from 1 to 54. The mean of center provider KAP score was 72% (SD = 10%) while that of quality of care score was 59% (SD = 11%). The characteristics of the centers and center providers are presented by type of center in Table 2.

**Table 2:** Summary statistics of childcare centers and center providers by type of center.

Variable	Level	Home-based (*N* = 45)	Center-based (*N* = 14)	Faith-based (*N* = 7)	Total (*N* = 66)
Caregiver sex	Male	0 (0%)	2 (14%)	1 (14%)	3 (5)
	Female	45 (100%)	12 (86%)	6 (86%)	63 (95)
Caregiver Age (years)	Mean (SD)	40 (9)	39 (10)	44 (14)	40.2 (10.0)
	Range	[23–74]	[23–57]	[24–63]	[23–74]
Highest education level completed	Primary	27 (60%)	2 (14%)	1 (14%)	30(45)
	Secondary+	18 (40%)	12 (86%)	6 (86%)	36(55)
Location of childcare center	Korogocho	7 (16%)	5 (36%)	3 (43%)	15 (23)
	Viwandani	38 (84%)	9 (64%)	4 (57%)	51 (77)
Provider trained in ECD		8 (18%)	9 (64%)	5 (71%)	22(33%)
Number of caregivers in center	Median (IQR)	1 (1–1)	2 (1–2)	1 (1–2)	1(1–2)
	Range	[1–4]	[1–3]	[1–2]	[1–4]
Number of children in center	Median (IQR)	7 (4–10)	26 (15–36)	33 (20–54)	10(5–20)
	Range	[1–25]	[2–68]	[11–62]	[1–68]
Caregiver: child ratio	Median (IQR)	7 (4–8)	14 (9–22)	21 (11–53)	8 (5–13)
	Range	[1–20]	[2–30]	[10–54]	[1–54]
Years of operation (median, IQR, and range)	Median (IQR)	2 (1–7)	4 (2–10)	4 (3–14)	3.5 (1–8)
	Range	[0–22]	[0–27]	[1–21]	[0–27]
Receive support from any organization		4 (9%)	2 (14%)	1 (14%)	7(11%)
Center provider KAP score (percentage of correct responses)	Mean (SD)	69 (9)	78 (9)	75 (7)	72 (10)
	Range	[47–85]	[63–94]	[65–87]	[47–94]
Quality of childcare score (percentage of correct responses)	Mean (SD)	55 (10)	65 (11)	70 (3)	59 (11)
	Range	[35–77]	[48–87]	[67–74]	[35–87]

### Correlations between predictor variables

Correlation analysis between numerical and ordinal predictor variables revealed that the predictors were weakly correlated with each other; all correlation coefficients (Spearman’s *ρ*) were less than 0.5. The correlation between center provider knowledge score and their education level was *ρ* = 0.228, showing that center provider knowledge was weakly related to the level of education. The correlation matrix is shown in Table 3.

**Table 3:** Spearman’s rank correlation coefficients between predictor variables.

Variables	Knowledge score	provider age	Education level	Caregiver-child ratio	Years of operation
Knowledge score	1.000				
Provider age	−0.019	1.000			
Education level	0.228	−0.089	1.000		
Caregiver–child ratio	0.251	0.142	0.392	1.000	
Years of operation	−0.181	0.152	0.047	0.334	1.000

### Internal consistency of the tools and interrater reliability

Four field interviewers took part in the administration of the questionnaires to the center providers and the quality assessments through observation. We used Cronbach’s alpha to measure the internal consistency of the quality tool. This was done for data collected by each of the observers. Generally, there was a high level of internal consistency ranging from 0.81 alpha to 0.86 alpha, as shown in Appendix 3. There was no reliability check for the observers because paired observations were not possible during the COVID-19 restrictions when data collection was done.

### Quality of childcare center score

**Figure 5:**
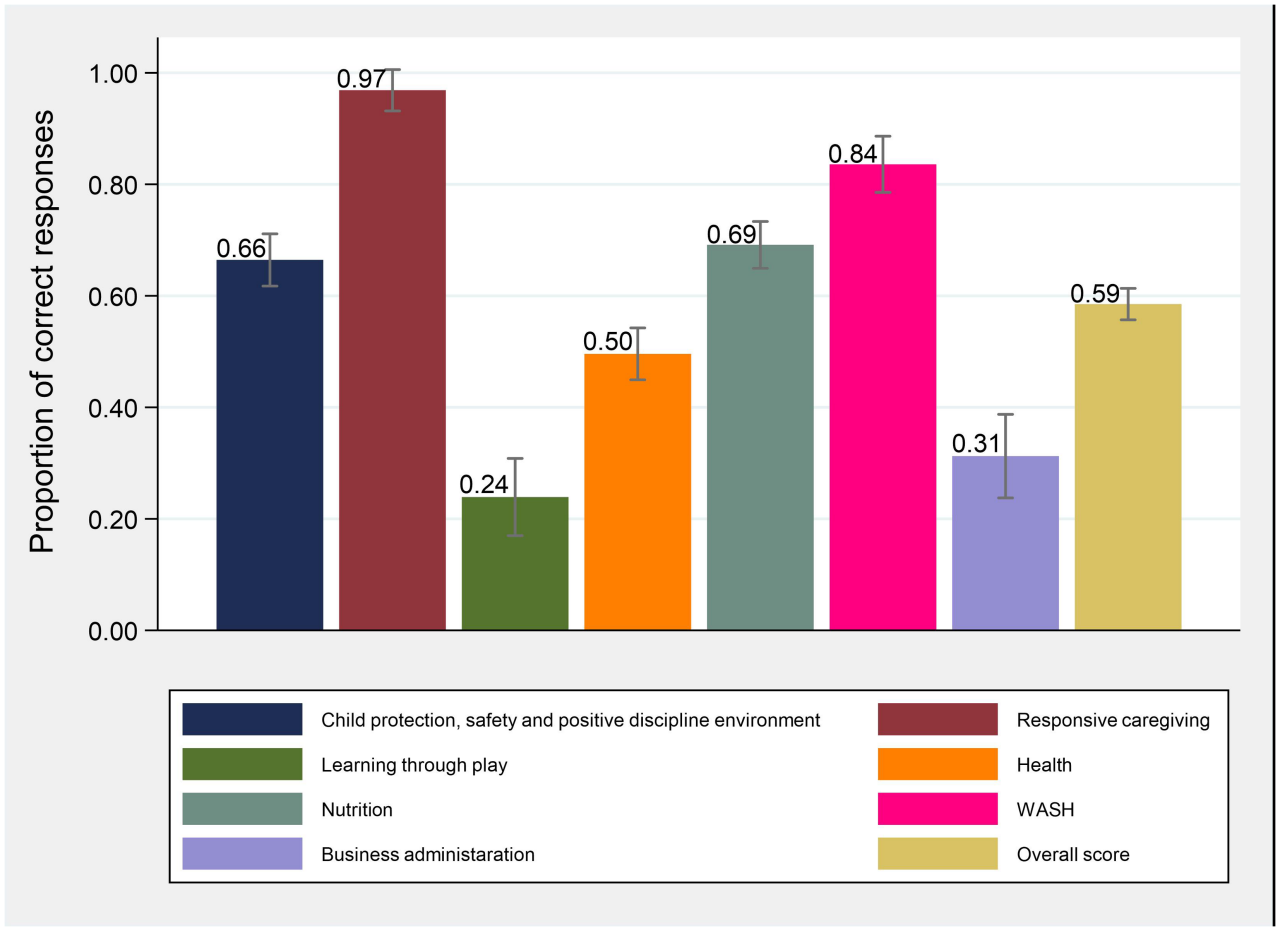
Average quality of care score across different components (mean (95% CI) proportions).

There were 31 items under childcare center quality each assigned one point for a correct response (Appendix 1). The score was given in terms of percentage; hence, the highest possible score was 100 (i.e., 31 points). The overall mean quality score was 59% (95% CI: 56, 61). The childcare centers had the highest score in responsive caregiving (97%; 95% CI: 93, 100) and the least score in learning through play (24%; 95% CI: 17, 31). They had above-average scores in four components (responsive caregiving, WASH, nutrition, and child protection and safe environment) and below-average score in three components (learning through play, business administration, and health) (Figure 5).

### Distribution of the quality of childcare center score based on potential predictors

#### Categorical potential predictors

**Figure 6:**
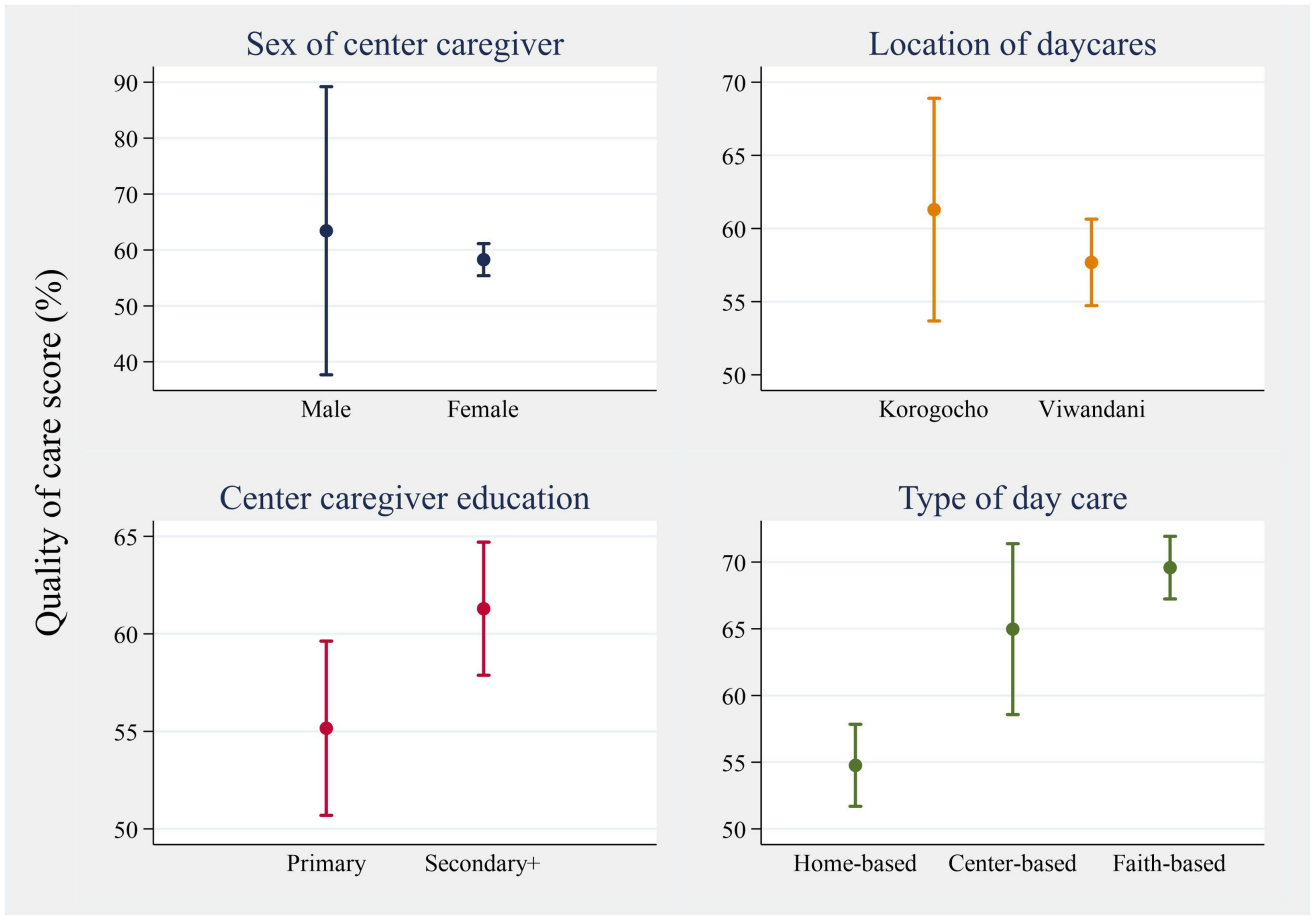
Mean (95% CI) of the quality of care score across categorical predictors.

The mean quality of care score was similar, regardless of sex [men: 63.4; women: 58.3; *p* = 0.155] and location (*p* = 0.344), while it was significantly different between levels of education (*p* = 0.030) and type of childcare center (home-based vs. center-based: *p* = 0.003; home-based vs. faith-based: *p* < 0.001) (Figure 6). In Korogocho, the mean score was 61% (95% CI: 54, 68), while in Viwandani, it was 58% (95% CI: 55, 60). The mean scores in the two locations were not significantly different. Center caregivers with primary education had a mean of 55% (95% CI: 51, 60) while those with secondary and above had 61% (95% CI: 58, 65). Regarding the type of childcare center, faith-based centers had the highest mean score [70% (95% CI: 68, 72)], followed by center-based [65% (95% CI: 59, 71)] and, lastly, home-based [55% (95% CI: 52, 58)].

#### Continuous potential predictors

**Figure 7:**
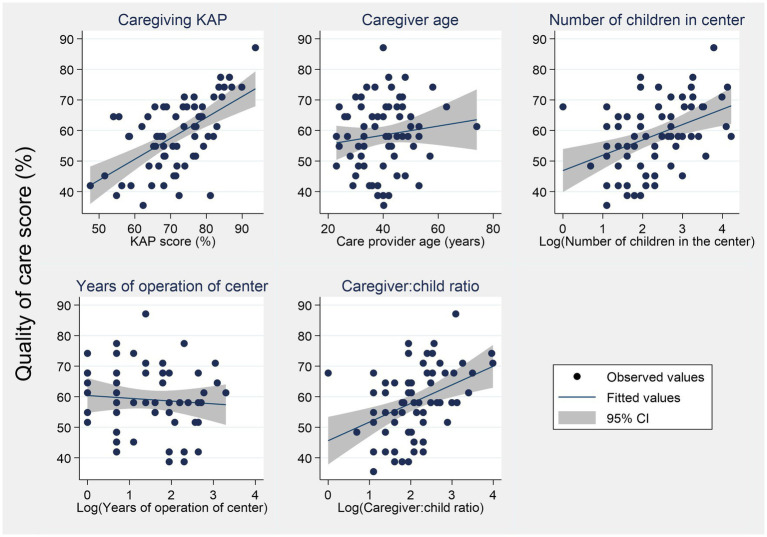
Distribution of the quality of care score across continuous outcomes.

The continuous predictors were plotted against the quality of care score to illustrate the magnitude and direction of their relationship (Figure 7). The number of children in the center, years of operation of the center, and the ratio of children: caregiver had non-linear relationships with the outcome variable (quality of care score). As a remedy, these three variables were log-transformed. There was a moderate correlation between the quality of care score and the three potential predictors, namely, caregivers’ KAP (*r* = 0.586), the natural logarithm of the number of children in center (*r* = 0.409), and the natural logarithm (ln) of the ratio of children: caregiver (*r* = 0.402). The two other predictors had weak correlations with the quality of care score: caregiver age (*r* = 0.131) and the natural logarithm years of operation of center (*r* = −0.076).

### Crude associations between center caregiver and childcare center characteristics, with quality of center score

Significant individual predictors included education level, type of center, whether center received some form of support, the ratio of the number of children to one caregiver, number of children in the center, and center provider KAP score.

The mean quality score of center caregivers with at least secondary education was 6.13% higher than that of caregivers with primary education ( β = 6.13; 95% CI: 0.63, 11.63). For every 1% increase in the caregiver’s KAP score, the quality of care score increased by 0.68% ( β = 0.68; 95% CI: 0.44, 0.93) (see Table 4).

**Table 4:** Crude association between the quality of care score and the center caregiver and childcare center characteristics.

Variable	Level/statistic	Mean (SD)/Correlation	Crude association	
			β -Coefficient (95% CI)	Value of *p*
Caregiver sex	Male	63.4 (±10.4)	ND	ND
	Female	58.3 (±11.4)		
Caregiver Age (years)	Correlation^a^	0.131	0.15 [−0.06, 0.35]	0.155
Highest education level completed	Primary	55.2 (±12.0)	Ref.	
	Secondary+	61.3 (±10.1)	6.13 [0.63, 11.63]	**0.030**
Location of childcare center	Korogocho	61.3 (±13.7)	Ref.	
	Viwandani	57.7 (±10.5)	−3.61 [−11.16, 3.95]	0.344
Type of childcare center	Home-based	54.8 (±10.2)	Ref.	
	Center-based	65.0 (±11.1)	10.20 [3.60, 16.82]	**0.003**
	Faith-based	69.6 (±2.5)	14.82 [11.24, 18.40]	**<0.001**
Center received some form of support	No	57.7 (±11.6)	7.24 [1.53, 12.95]	**0.014**
	Yes	65.0 (±6.8)		
Log (Caregiver: child ratio)	Correlation^a^	0.402	6.09	**0.002**
			[2.41, 9.77]	
Log (number of children in center)	Correlation^a^	0.409	4.97 [2.00, 7.95]	**0.001**
Log (years of operation)	Correlation^a^	−0.076	−0.91 [−3.73, 1.91]	0.520
Center provider KAP scores (%)	Correlation^a^	0.586	0.68 [0.44, 0.93]	**<0.001**

ND – Association not done due to small count in one cell (men); ^a^Pearson’s correlation.

The bold statistics represent those factors that are significantly associated with quality of childcare centers.

### Adjusted associations between the center caregiver and childcare center characteristics with center quality score

A multiple linear regression model was used to determine the adjusted effect of the predictors on the quality of care score. The regression model was statistically significant (*p* < 0.001), implying that the model can statistically significantly predict the outcome (quality of childcare center score). The adjusted *R*^2^ = 0.475, indicates that our model explains 47.5% of the variation in the quality of childcare center score in the study population (Table 5).

**Table 5:** Adjusted association between quality of childcare center and potential predictors.

Variable	Level	Adjusted association		
		β -Coefficient	[95% CI]	Value of *p*
Caregiver Age (years)	Mean	0.10	[−0.09, 0.30]	0.299
Highest education level completed	Primary	Reference		
	Secondary+	1.55	[−3.08, 6.19]	0.504
Location of childcare center	Korogocho	Reference		
	Viwandani	0.77	[−4.73, 6.28]	0.779
Type of childcare center	Home-based	Reference		
	Center-based	3.65	[−2.57, 9.87]	0.245
	Faith-based	**8.68**	**[2.32, 15.04]**	**0.008**
Center received some form of support	No	Reference		
	Yes	3.65	[−2.51, 9.81]	0.240
Children: caregiver ratio	Mean	1.01	[−2.98, 5.00]	0.614
Center provider KAP scores	Mean	**0.51**	**[0.18, 0.84]**	**0.003**
Field interviewer	Interviewer 1	Reference		
	Interviewer 2	−0.20	[−5.62, 5.22]	0.942
	Interviewer 3	−2.01	[−7.70, 3.68]	0.481
	Interviewer 4	−2.04	[−8.56, 4.47]	0.532
Model summary statistics				
Mean dependent variables	58.504	SD dependent variable	11.315	
*R*-squared	0.475	Number of observations	66	
*F*-test	7.834	Prob > F	<0.001	

The bold statistics represent those factors that are significantly associated with quality of childcare centers.

The type of childcare center (faith-based vs. home-based: *p* = 0.008) and the center provider KAP score (*p* = 0.003) were statistically significantly associated with the quality of care score. After adjusting for the other factors in the model (Table 5), the mean quality of childcare center score of faith-based centers was 9.2% points higher than that of home-based centers ( β = 8.68; 95% CI: 2.32, 15.04). In other words, faith-based centers provided better quality childcare services than home-based centers.

Controlling for other predictors in the model (including the interviewer effect), for every 1% increase in the caregiver KAP score, the mean quality of childcare center score increased by 0.51% ( β = 0.51; 95% CI: 0.18, 0.84). This implies that there was a positive association between the KAP score and the quality of childcare center score, that is, the higher the KAP score, the higher the quality score (Table 5). The interviewer effect was not significantly associated with the quality of center score.

### Predicted values of quality score across significant factors

**Figure 8:**
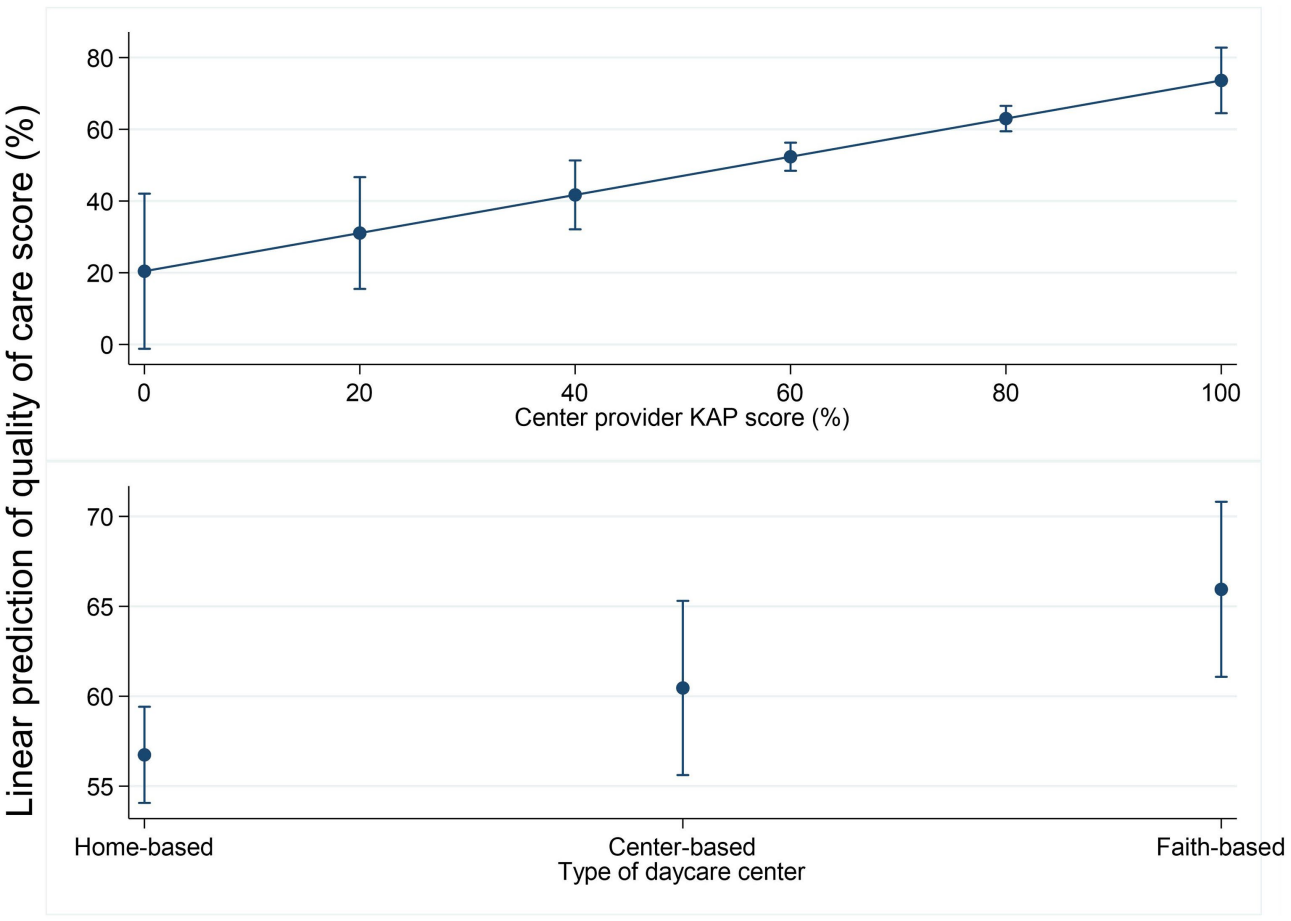
Linear prediction of the quality of care by center provider KAP score and center type.

The predicted values of the quality of care score increased consistently with increasing center provider KAP score (Figure 8). Faith-based centers had the highest mean predicted quality score (66%), followed by the center-based (60%) and, lastly, home-based (57%) centers.

## Discussion

The current study examined profiles and predictors of the quality of childcare centers in two slum communities (Korogocho and Viwandani) in Nairobi, Kenya, ahead of the implementation of a co-designed intervention, that aimed to improve the quality of childcare centers. We found a relatively high concentration of informal childcare centers in the two informal settlements, the majority of which were home-based centers, particularly in Viwandani, which is closest to the industrial areas of Nairobi. This is consistent with an earlier report indicating that 46% of employed and 23% of unemployed parents in the slums (Korogocho) use paid childcare as the primary childcare strategy (31). The high proportion of women in this location working outside the home (31, 32) explains this high demand and mushrooming of informal childcare centers. Our data highlight the low level of support or training received by the providers, the majority of whom are women, running these centers. Similar quality issues have been reported in studies that have examined the provision of childcare within similar urban settlements in Africa (33, 34).

The data show generally moderate quality scores (mean = 59%) across the types of centers, especially among home-based childcare centers (barely at a score of 55%) which were the majority. The results also reveal two major predictors of quality, i.e., center caregiver knowledge and practice (KAP) score and type of childcare center. Higher scores of center provider KAP were significantly associated with higher scores on center quality, while home-based childcare centers were associated with the lowest quality of care, followed by center-based and faith-based daycare centers. Other characteristics including provider education, location of center, and child-to-caregiver ratio showed significant crude correlations with the center quality; however, these diminished after multivariable analysis. Given the low quality of centers, an intervention that promotes the standard of care for optimum child growth and development is critical (35).

The finding that center provider knowledge and practices and type of center significantly predicted quality indicates that these two factors should be targeted in improving the standards of childcare facilities in this setting. The poor skills of center providers observed could be attributed to a lack of training in childcare or ECD. Furthermore, the majority of the centers were not registered, which, as reported in a related intervention development paper (36), was in part because of the lack of qualification required for registration.

While the quality of childcare center environment across the different types of centers was generally poor, home-based childcare centers were markedly poor in most of the aspects and seem to be a major driver of the overall quality score since they formed a greater proportion of the centers. The poor quality of home-based centers would be expected; since from our experience and interaction with the providers, the center managers/providers were generally not trained and did not receive any external support to run the daycare, e.g., from the government or NGOs. Home-based centers were usually established informally by a community member who volunteers to help out another woman who wants to join paid work but has no one to take care of her child. Gradually, more and more women approach the volunteer and the number of children under her care grows to become a fully-fledged childcare center within her household. While such centers respond to the need for childcare in the community, they provide substandard facilities characterized by small, dark spaces with limited or no play area, poor WASH facilities, lack play materials, and are run by providers who are neither trained nor experienced, thus putting the children under their care at risk of poor health, growth, and development (35).

The findings of this study contribute new evidence to the currently small but growing evidence on childcare centers in LMICs, particularly on the quality of childcare centers and key drivers of the poor standards of childcare centers in the informal settlements in Nairobi. These findings indicate the need for interventions to improve childcare services in these low-income settings by addressing the lack of skills of center providers and other factors contributing to the poor childcare environment. Furthermore, the findings extend our understanding of factors that determine the quality of childcare services and are consistent with the findings from previous studies. For instance, caregiver practices, particularly their interactions with children, have been reported to affect the quality of childcare services in South Korea (26) and other low-income settings in the US, South America, and Europe (37). In other studies, e.g., a study by Ghazvini and Mullis (28), the role of specialized caregiver training, higher adult–child ratios, and use of planned activities have been reported as best predictors of higher quality care and sensitive caregiver–child interaction in centers. However, in our study, the effect of the care provider and child ratio diminished significantly when we adjusted for other factors.

## Limitations

Our study had some limitations. The center provider KAP score was a global variable derived from summing several items and used as a summative score in the analysis of the association with center quality. Given the small sample size, it was not possible to examine associations with individual items or conduct factor analysis to identify the most important components of caregiver KAP. Similarly, within center type, there should be specific characteristics that are most critical; however, adjusted regressions with the number of children in the center, caregiver–child ratio, and years of operation were not statistically significant. This might also be obscured by the small sample size, and hence, further studies with a large sample size may reveal the most critical characteristics of the center environment. The mapping and assessments were done within the context of the COVID-19 pandemic and therefore may have missed some of the childcare centers that were closed temporarily due to the restrictions on schools at that time. The cross-sectional design used to assess the predictors at baseline provides useful insights into the associations between the quality of daycare and other factors; however, it does not infer causal effect direction. Finally, we acknowledge the absence of a reliability check on the observers because paired observations were not possible during the COVID-19 restrictions when data collection was carried out.

## Conclusion

Although conditions are poor and education levels of childcare center providers are low, improvements in quality are possible with interventions that can increase knowledge and skills. It is, therefore, important that programs that aim to improve the quality of childcare in such settings focus on training and support supervision of center providers.

### Recommendations for policy, practice, and research

Based on the findings of this study, we recommend training and support supervision of childcare center providers with priority to home-based centers to enhance their capacity to provide quality care for the children. We recommend larger studies employing experimental designs to examine the influence of several factors and identify the most important aspects of center provider KAP in determining the quality of center environment.

## Data availability statement

The raw data supporting the conclusions of this article will be made available by the authors, without undue reservation.

## Ethics statement

The studies involving humans were approved by Amref Health Africa’s Ethics and Scientific Review Committee ESRC, Kenya (Ref: P7802020 on 20th April 2020) and from the University of York (Ref: HSRGC 20th March 2020). The studies were conducted in accordance with the local legislation and institutional requirements. The participants provided their written informed consent to participate in this study.

## Author contributions

MN co-led funding acquisition, study conceptualization, investigation, methodology, manuscript preparation, project administration, supervision, participated in data curation, formal analysis, reviewed, and edited the manuscript. NL led data analysis, curation, participated in project administration, and manuscript writing. LO participated in the project administration, data curation, analysis, and writing. KO participated in the conceptualization, investigation, methodology of the study, project administration, data curation, formal analysis, and writing review and editing. RM participated in project administration, data curation, and writing review and editing of the manuscript. MA-O participated in project administration, data curation, and writing review and editing of the manuscript. PK-W participated in the conceptualization, investigation, methodology, project administration, data curation, and writing review and editing of the manuscript. EK-M contributed to the design, supported project administration, and writing review and editing of the manuscript. HE led the acquisition of funding, participated in the conceptualization, investigation, methodology, contributed to project administration, supervision, analysis, and writing of the manuscript. MN, NL, LO, MK, PA, KO, RM, MA-O, PK-W, EK-M, and HE participated in the writing, reviewing, and editing of the final version of the manuscript. All authors contributed to the article and approved the submitted version.
