# Supplementary material for: Predictors of quality of childcare centers in low-income settings: findings from a cross-sectional study in two Nairobi slums
Source: Front Public Health. 2023 Oct 31;11:1163491. doi: 10.3389/fpubh.2023.1163491 (PMC10644197; doi:10.3389/fpubh.2023.1163491)
Supplement: Supplementary file 1 [file Data_Sheet_1.docx]

**Appendix 1: Quality of daycare center (center environment) scoring guide**

| **Component** | **Item** | **Responses** | **Score of response** | |
| --- | --- | --- | --- | --- |
|  |  |  | **0** | **1** |
| **Child protection, Child safety, Child abuse, & positive discipline**  **Stimulating Environment** | Safe & free of obvious hazards (e.g. sharps, fire, ditches, live wires, collapsing walls, hot water, medicines, small beads/beans) | 1-Yes  2-No | 2 | 1 |
|  | Sufficient lighting & ventilation (room not dark) |  |  |  |
|  | At least one mat / carpet |  |  |  |
|  | Suitable indoor space for the number of children (one child per sq. Meter) |  |  |  |
|  | Children look happy and calm (e.g. not looking irritable, grumpy , crying, nervous, or/and depressed) |  |  |  |
|  | Comfortable outdoor space (spacious, not crowded, dry, and flat) |  |  |  |
|  | No instance of corporal punishment observed (i.e. yelling, hitting, pinching, other physical punishment) |  |  |  |
| ***Maximum component raw score = 7; Percent score = (Raw score/7)*100%*** | | | | |
| **Responsive Caregivers** | Fifteen children (or less) per caregiver | 1-Yes  2-No | 2 | 1 |
|  | Caregiver is aware of all children and attends to their needs(knows about each child individually and their needs) |  |  |  |
| ***Maximum component raw score = 2; Percent score = (Raw score/2)*100%*** | | | | |
| **Learning through Play** | Each child has something to play with | 1-Yes  2-No | 2 | 1 |
|  | Daily routine posted & used |  |  |  |
|  | One learning centre present & labelled (separate area with play materials, toys, books etc. for learning) |  |  |  |
| ***Maximum component raw score = 3; Percent score = (Raw score/3)*100%*** | | | | |
| **Health** | Conducts daily health check and understands what to do if a child is sick | 1-Yes  2-No | 2 | 1 |
|  | First aid kit & knowledge on use |  |  |  |
|  | Immunization status of children tracked |  |  |  |
|  | Evidence for temperature recording (thermometer, temp records available) |  |  |  |
|  | ***Evidence for protection against Covid 19:*** |  |  |  |
|  | Handwashing station (clean water and soap) present |  |  |  |
|  | Sanitizer present |  |  |  |
|  | Masks present |  |  |  |
| ***Maximum component raw score = 7; Percent score = (Raw score/7)*100%*** | | | | |
| **Nutrition** | Children receive morning *uji* (porridge) | 1-Yes  2-No | 2 | 1 |
|  | Children receive lunch |  |  |  |
|  | Children are served with warm food |  |  |  |
|  | Poster of what a balanced diet is, is displayed |  |  |  |
| ***Maximum component raw score = 4; Percent score = (Raw score/4)*100%*** | | | | |
| **Water, Sanitation & Hygiene (WASH)** | Hand washing facility with soap available and in use | 1-Yes  2-No | 2 | 1 |
|  | At least one potty for every 5 children |  |  |  |
|  | Centre is cleaned daily (centre is visibly clean) |  |  |  |
|  | Access to clean and safe drinking water |  |  |  |
| ***Maximum component raw score = 4; Percent score = (Raw score/4)*100%*** | | | | |
| **Business & Administration** | Daily attendance recording available | 1-Yes  2-No | 2 | 1 |
|  | Track finances daily/ weekly / monthly (record available) |  |  |  |
|  | Centre policies clearly posted(any information on how their centre is run/managed) |  |  |  |
|  | Budget available |  |  |  |
| ***Maximum component raw score = 4; Percent score = (Raw score/4)*100%*** | | | | |
|  | | | | |
| **Maximum total center quality raw score = 7+2+3+7+4+4+4 = 31;**  **Overall center quality percent score = Mean of component percent scores (sum of all component percent scores/7)** | | | | |

**Appendix 2: Centre provider KAPs scoring guide**

| **Component** | **Item** | **Responses** | **Scoring of response** | |
| --- | --- | --- | --- | --- |
|  |  |  | **0** | **1** |
| **Knowledge on Business management** | It is important to develop the budget for the day-care centre at the beginning of the term | 1 - Agree completely 2 - Agree a little 3 - Disagree a little 4 - Disagree completely | 3,4 | 1,2 |
|  | It is important to record which parent has paid |  |  |  |
|  | It is important to record how much each parent has paid |  |  |  |
|  | Do you/centre owner track the income and expenses made in your centre? | 1-Yes  2-No | 2 | 1 |
|  | Do you make or prepare a budget for your centre at the beginning of the week/month? |  |  |  |
|  | Do you have the centre policy on pricing and opening times for the centre? (Centre name, operating hrs, fees, and caregiver contacts).  *Ask the centre provider if it is not visible* |  |  |  |
|  | Do you track/keep accounts for your centre showing income and expenditure? |  |  |  |
|  | Do you track attendance daily? |  |  |  |
|  | Is the business license posted? |  |  |  |
|  | Do you have an attendance register showing how many children come to your centre per day? |  |  |  |
| ***Maximum component raw score = 10; Percent score = (Raw score/10)*100%*** | | | | |
| **Knowledge on Safety** | It is important to always ensure a safe environment for the children | 1 - Agree completely 2 - Agree a little 3 - Disagree a little 4 - Disagree completely | 3,4 | 1,2 |
|  | It is important for a caregiver or other people to keep a child in visual range and to look at him/her often |  |  |  |
|  | Centre providers should constantly watch out for and remove any potentially hazardous materials (e.g. sharps, fire, ditches, live wires, collapsing walls, hot water, medicines, small beads/beans) |  |  |  |
| ***Maximum component raw score = 3; Percent score = (Raw score/3)*100%*** | | | | |
| **Knowledge on responsive caregiving/discipline** | Children must be handled harshly for them to develop better. | 1-Always  2-Sometimes  3-Never | 1,2 | 3 |
|  | Which method do you use most often to deal with children when they misbehave?  *(tick only one option)* | 1-Physical punishment  2-Verbal punishment  3-Distract child with another activity  4-Explains wrong deeds to child calmly | 1,2,3 | 4 |
|  | In the past 2 weeks, how many times have children been physically punished in the centre? *(pinch/slap/spanked )* | 1-Daily  2-Once to twice in a week  3-Never | 1,2 | 3 |
| ***Maximum component raw score = 3; Percent score = 3(Raw score/3)*100%*** | | | | |
| **Learning through play** | It is important for children to play | 1 - Agree completely 2 - Agree a little 3 - Disagree a little 4 - Disagree completely | 3,4 | 1,2 |
|  | Does each child get an opportunity to play with toy or something | 1-Yes  2-No | 2 | 1 |
|  | Do you post the Children’s work on the wall |  |  |  |
| ***Maximum component raw score = 3; Percent score = (Raw score/3)*100%*** | | | | |
| **Knowledge on health** | It is important to always take a child’s temperature on arrival at the day care | 1 - Agree completely 2 - Agree a little 3 - Disagree a little 4 - Disagree completely | 3,4 | 1,2 |
|  | Conduct daily health checks? | 1-Yes  2-No | 2 | 1 |
|  | Do you understand what to do if a child is sick? |  |  |  |
|  | Do you know if the children you care for have been immunized? | 1-Yes for all  2-Yes for some  3-No | 3 | 1,2 |
|  | Can you tell me which vaccinations children should have and when? (*Interviewer confirms if answer given by centre provider is correct or wrong*) |  |  |  |
|  | Birth (BCG & Polio 0) | 1-Correct  2-Wrong | 2 | 1 |
|  | six weeks (DPT and Polio 1) |  |  |  |
|  | Ten weeks(DPT2 and Polio 2) |  |  |  |
|  | Fourteen weeks (DPT3 and Polio 3) |  |  |  |
|  | Nine months (measles) |  |  |  |
|  | *(NB: Interviewer will have to know the immunization schedule and compare their answer to tick correct or wrong)* |  |  |  |
| ***Maximum component raw score = 9; Percent score = (Raw score/9)*100%*** | | | | |
| **Nutrition** | Children must be fed with a balanced diet to be healthy, and grow and develop well | 1 - Agree completely 2 - Agree a little 3 - Disagree a little 4 - Disagree completely | 3,4 | 1,2 |
|  | How often do you encourage and make sure children finish all the food on their plates: *(Tick only one)* | 1-Every meal  2-Often I’m too busy so can only do this sometimes  3-I never have time to do this  4-I think it is better to let children eat on their own | 2,3,4 | 1 |
|  | Do you plan and provide a menu with diverse foods for a day/week/month? | 1-Yes  2-No | 2 | 1 |
|  | If you don’t cook in your centre, do you advise parents on the foods to give their children to bring with them? |  |  |  |
|  | Do you feel confident telling them which foods are appropriate? |  |  |  |
| ***Maximum component raw score = 5; Percent score = (Raw score/5)*100%*** | | | | |
| **Water, Sanitation & Hygiene (WASH)** | When do you do handwashing in the daycare centre? *(tick or cross against each item)* |  |  |  |
|  | Before preparing meals | 1-Yes  2-No | 2 | 1 |
|  | After changing diapers |  |  |  |
|  | After handling soiled toys |  |  |  |
|  | After visiting the toilet |  |  |  |
|  | How do you do your handwashing? | 1-With water only  2-With water and soap  3-No handwashing | 1,3 | 2 |
|  | How often do you clean the centre? | 1-Once a day  2-More than once a day  3-Every other day | 1,3 | 2 |
|  | What type of drinking water do you use? | 1-Unboiled water  2-Boiled water  3-Water treated with water guard  4-Bottled water | 1 | 2,3,4 |
|  | How do you dispose of your potty/ diapers waste? | 1-In the trench  2-Toilet  3-Dustbin  4-Pit latrine  5-Hole dug | 1,2,3 | 4,5 |
| ***Maximum component raw score = 8; Percent score = (Raw score/8)*100%*** | | | | |
|  | | | | |
| **Maximum total centre provider KAPs raw score = 10+3+3+3+9+5+8 = 41;**  **Overall centre provider KAPs percent score = Mean of component percent scores (sum of all component percent scores/7)** | | | | |

**Appendix 3: Internal consistency of the quality tool**

| **Interviewer** | **Cronbach’s alpha** | **95% CI** |
| --- | --- | --- |
| Interviewer 1 | 0.84 | [0.78, 0.87] |
| Interviewer 2 | 0.81 | [0.76, 0.86] |
| Interviewer 3 | 0.86 | [0.76, 0.92] |
| Interviewer 4 | 0.84 | [0.81, 0.89] |
